# Effect of Coenzyme Q10 on early wound healing after recession coverage surgery with the modified coronally advanced tunnel technique and a connective tissue graft: A 6-month, triple-blinded, randomized, placebo-controlled pilot trial

**DOI:** 10.1007/s00784-024-05790-4

**Published:** 2024-07-11

**Authors:** Alexandra Stähli, Siro P. De Ry, Andrea Roccuzzo, Jean-Claude Imber, Anton Sculean

**Affiliations:** Department of Periodontology, School of Dental Medicine, University of Bern, Freiburgstrasse 7, CH-3010 Berne, Switzerland

**Keywords:** Coenzyme Q10, Wound healing, Modified coronally advanced tunnel technique, Connective tissue graft

## Abstract

**Objectives:**

Coenzyme Q10 (CoQ10) or ubiquinone is one of a cell’s most important electron carriers during oxidative phosphorylation and many other cellular processes. As a strong anti-oxidant with further anti-inflammatory effects CoQ10 is of potential therapeutical value. The aim of this randomized controlled clinical trial was to investigate the effect of topical CoQ10 on early wound healing after recession coverage surgery using the modified coronally advanced tunnel (MCAT) and palatal connective tissue graft (CTG).

**Materials and methods:**

Thirty patients with buccal gingival recessions were evaluated after being randomly allocated to: 1) MCAT and CTG with topical application of a coenzyme Q10 spray for 21 days or 2) MCAT and CTG with placebo spray. Wound healing was evaluated by the early wound healing index (EHI). Patient-reported pain was analyzed by a 100-mm visual analogue scale (VAS) at day 2, 7, 14 and 21 post-surgically. Mean recession coverage, gain of keratinized tissue and esthetic outcomes were assessed at 6 months.

**Results:**

EHI and pain scores showed no significant differences. Time to recovery defined as VAS<10 mm was shorter in the test group. Mean root coverage after 6 months was 84.62 ± 26.57% and 72.19 ± 26.30% for test and placebo, p=0.052. Complete root coverage was obtained in 9 (60%) test and in 2 (13.3%) placebo patients. Increase in keratinized tissue width and esthetical outcomes were similar for both groups.

**Conclusion:**

CoQ10 had no significant effect on early wound healing and on mean root coverage after 6 months.

**Clinical relevance:**

Early wound healing: in young healthy patients with no inflammatory oral conditions topical CoQ10 does not improve early healing.

**Supplementary Information:**

The online version contains supplementary material available at 10.1007/s00784-024-05790-4.

## Introduction

Gingival recessions constitute a highly prevalent clinical condition, defined as the displacement of the soft-tissue margin apical to the cemento-enamel junction [1, 2]. Besides aesthetic concerns, gingival recessions may be associated with root hypersensitivity, impaired oral hygiene, and progressing attachment loss [3]. When left untreated, gingival recessions may progress further, even in patients with adequate oral hygiene [4]. Data from a systematic review revealed that 78.1% of sites with gingival recession at baseline worsened during a 2-year follow-up period, while 79.3% of patients showed an increase in the number of recessions [4].

At present, in the great majority of patients, gingival recessions are treated with a subepithelial connective tissue graft (SCTG) in conjunction with either a coronally advanced flap (CAF) or a coronally advanced tunnel, both of which are considered state of the art. Today, the routine postoperative protocol often includes 3-7 days of antibiotic treatment to prevent postoperative graft infection and to relieve symptoms [1–4]. However, antibiotic treatment should be prescribed with caution due to increasing bacterial resistance worldwide and potential systemic side effects in individual patients [5–7]. Therefore, there is a need to search for novel substances that can positively influence early wound healing after various types of regenerative surgical procedures [5–7] .

Coenzyme Q10 (CoQ10), an endogenously produced molecule, may offer such an approach. CoQ10, also known as ubiquinone because of its ubiquitous presence in cells, acts as an electron carrier in the generation of adenosine triphosphate along the inner mitochondrial cell membrane [8]. CoQ10 also accepts electrons from numerous other enzymatic reactions, linking oxidative phosphorylation, fatty acid metabolism and glycolysis [3–7]. CoQ10 also has anti-inflammatory effects by inhibiting the translocation of nuclear factor kappa B (NF-κB) into the nucleus, a central transcription factor for inflammation-related genes [8]. Over the last decade our understanding of CoQ10 has expanded with an increasing number of functions emerging in processes like gene modulation, mitochondrial homeostasis, cell signaling, insulin metabolism, or senescence itself [9–11]. However, it is mostly due to its anti-oxidative and anti-inflammatory properties that CoQ10 might be of therapeutical interest for numerous kinds of surgical interventions and chronic diseases.

With regard to oral wound healing, topical application of CoQ10 after tooth extraction in rats was associated with higher collagen density and decreased levels of polymorphonuclear leukocytes, interleukin -1β, tumor-necrosis factor-α, and NF-κB as compared to the control group [12]. A recent review reported on a beneficial effect of the application of CoQ10 in combination with scaling and root planing (SRP) during non-surgical periodontal therapy: the test group showed a significantly greater reduction of probing depth, bleeding on probing and plaque scores compared to SRP alone [13].

To improve wound healing many other agents or naturally occurring substances such as turmeric, green tea, flavonoids, or quercetin have been investigated for their beneficial effects [14]. All of these substances anti-inflammatory and anti-oxidant effects have been attributed. For example, resveratrol, which is abundant in red wine, increased bone formation in tooth extraction sites, cell proliferation and alkaline phosphatase (ALP) activity in the mouse model [15]. Similarly, another strategy has been the application of platelet concentrates or platelet rich fibrin (PRF) to the wound area. Platelets and leukocytes entrapped within a fibrin mesh provide the slow release of growth factors and antibacterial activity [16, 17]. When using CAF and PRF a significantly greater relative recession coverage and improved clinical attachment level were obtained than with CAF alone.

The aim of this randomized, placebo-controlled trial was to investigate the effect of topical coenzyme Q10 application on early wound healing and mean root coverage 6 months after recession coverage using a modified coronally advanced tunnel in conjunction with a connective tissue graft.

## Materials and methods

### Study design and patient selection

This study was designed as randomized, placebo-controlled, triple-blinded clinical pilot trial including two parallel groups (see CONSORT flow diagram, Supplement Figure). The study has been approved by the human subjects ethical board of the Kanton Bern in Switzerland (ID 2019-01542) and was conducted in accordance with the Helsinki Declaration of 1975, as revised in 2013 and is reported in compliance with the STROBE statement [18]. The study has been prospectively registered at ClinicalTrials.gov (NCT04487652).

Patients were included based on the following inclusion criteria:

- aged ≥ 18 years
- presence of ≥ 1 buccal gingival recessions (Recession type 1 (RT1) and 2 (RT2) according the Cairo classification [19, 20]
- signed informed consent

The following exclusion criteria were applied:

- systemic disease that compromises wound healing or hemostasis
- smoking > 5 cigarettes per day
- poor oral hygiene (i.e., FMPS > 20%)
- pregnancy or lactating at the date of inclusion
- previously performed periodontal surgery on the affected tooth

### Randomization

Randomization was performed by the spray supplier (Dr. med. Schütze GmbH, Attnang-Puchheim, Austria) using a computerized randomization table. The sprays were delivered in light-protecting bottles with consecutive numbers from 1 to 60. (The first patient received spray-bottle number 1 and 31, the second patient bottle number 2 and 32 and so on). Each patient received a dispenser to apply the spray and an oral and written instruction of how to apply the spray by the surgeon. The surgeon (A.S.) as well as the clinicians (S.DR.; A.St.; A.R.) who assessed the outcome measures were blinded to patient allocation. The list with the bottle randomization was in the hands of the spray supplier until data evaluation (ParoMit versus placebo, Dr. med. Schütze GmbH, Attnang-Puchheim, Austria).

### Surgical procedure

A total of 34 patients were consecutively operated using the MCAT technique including a connective tissue graft that was harvested from the palate [21]. All surgeries were performed by the same experienced surgeon (A.S.) according to the department’s protocol. More specifically, after root planing of the exposed root using Gracey curettes (Gracey Curettes, Stoma, Storz am Mark GmbH, Emmingen-Liptingen, Germany), an intrasulcular incision was performed with a microblade (Key Dent, Micro Blades, American Dental Systems) and for this purpose designed tunneling knives (Stoma Dentalsysteme GmbH&Co. KG, Emmingen-Liptingen, Germany). Following preparation of mucoperiosteal tunnel flap leaving the papillae untouched, the attaching muscles and attaching collagen fibers were released from the inner aspect of the flap with the microsurgical blades and curettes. In doing so, the tunneled flap would get tensionless and could be coronally advanced. Finally, the papillae were gently tunneled under using a specialized tunnel knife (Sculean-Aroca N°1, Stoma Dentalsysteme GmbH&Co. KG, Emmingen-Liptingen, Germany). Thereafter, a palatal connective tissue graft was harvested using the single incision technique [22]. Immediately thereafter the CTG was soaked in NaCl while closing the donor site with a modified mattress suture (5-0 Seralon, Serag-Wiessner). The CTG was pulled into the mucoperiosteal tunnel by means of single or mattress sutures and fixed at the inner side of the flap. Subsequently, the graft was fixed on the level of around 1 mm above the cemento-enamel-junction with a sling suture (6-0 Seralon, Serag-Wiessner GmbH&Co. KG, Naila, Germany). Finally, the tunnel flap was coronally moved in order to completely cover the graft and the exposed root. Fixation of the flap to the more coronal position was obtained by sling sutures [21, 23].

### Post-surgical procedure

Post-surgically patients were given analgesics (2x500 mg/day, mefenamic acid (Mephadolor, Mepha Pharma, Basel, Switzerland).

The palatal sutures were removed after 7 days whereas those of the recession site after 21 days postoperatively. Patients had to refrain from tooth brushing at the recession site until suture removal. In this time patients were frequently checked and cleaning of the surgical area was performed. Thereafter they were using an ultrasoft manual toothbrush (Paro surgical mega soft, Paro, Esro AG, Kirchberg ZH, Switzerland) employing the roll technique and after 1 month gradually returned to regular oral hygiene habits.

### Application of test or placebo spray

In the first post-operative week both wounds were sprayed 3 times a day applying 6 puffs on the recession and 4 puffs on the palatal site. Thereafter for the following 2 weeks, patients were asked to use the spray 3 times a day but only 3 puffs on the recession and 2 puffs on the palatal site. The test spray contained Kaneka Q10 (45 mg/ml) in a matrix of water, phospholipids and glycerin. The placebo spray contained no Kaneka Q10 but a coloring to mimic the test spray.

### Clinical parameters

The primary outcome was:

- the progress of wound healing assessed by the early wound healing index EHI previously defined by Wachtel et al. 2003 [24].

The secondary outcomes were:

- the assessment of patients’ postoperative comfort see VAS
- the assessment of keratinized tissue width (KTW) and gain of KTW
- the assessment mean root coverage (mRC) and complete root coverage (CRC) in %
- the assessment of the root coverage esthetic score (RES)

**Fig. 1:**
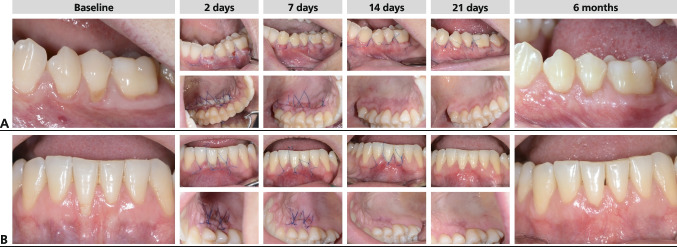
Representative cases illustrating early wound healing of test (**A**) and placebo sites (**B**). Early wound healing was clinically assessed at day 2, 7, 14, and 21 for both the donor and recipient site

The early wound healing index was chosen to clinically classify the sequelae of wound healing after periodontal surgery. According to Wachtel wound healing is quantified and graded into 5 stages. *Stage 1* is defined as healing without any fibrin layer. *Stage 2* comprises a thin fibrin line within the region of incision. In *stage 3* a pronounced fibrin coverage is detectable, *stage 4* describes an open wound including suppuration. Finally, a *stage 5* healing is characterized by pus formation. The index was assessed by clinical inspection and on photographs (Fig. 1).

Patient-reported pain outcomes were evaluated using a 100-mm visual analogue scale (VAS). Patients were asked to draw a line that represents their pain level for the time intervals 0-2 days, 2-7 days, 7-14 days, and 14-21 days postoperatively. Time to recovery was defined according to Tonetti when a VAS<10 mm was reached [25].

### Clinical parameters

The clinical parameters recession depth, recession depth reduction, keratinized tissue width (KTW), KTW gain, and probing depth (PD) were assessed at baseline and the 6-month visit. The percentage of mean root coverage (mRC) and complete root coverage (CRC) as well as the root coverage esthetic score (RES) were assessed at the final 6-month visit [26]. The RES takes 5 parameters into account. This score was evaluated based on the photographs.

#### Gingival margin (GM) level

Score 0: failure of root coverage (gingival margin apical or equal to the baseline recession), Score 3: partial root coverage, Score 6: complete root coverage with no detectable CEJ.

#### Marginal tissue contour (MTC)

Score 0: irregular gingival margin (it does not follow CEJ); Score 1: proper marginal contour/scalloped gingival margin (following CEJ).

#### Soft tissue texture (STT)

Score 0: presence of scar formation and/or keloid-like appearance; Score 1: absence of scar or keloid formation.

#### Mucogingival junction (MGJ)

Score 0: MGJ not aligned with MGJ on adjacent teeth; Score 1: MGJ aligned with MGJ on adjacent teeth.

#### Gingival color

Score 0: color of tissue differs from gingival color on adjacent teeth; Score 1: normal color and integration with the adjacent soft tissues.

The maximal score obtains 10 points. RES was assessed by two examiners on the photographs.

### Calibration

The outcome measurements were assessed by three calibrated clinicians (A.St., S.DR.; A.R.) who were blinded to the treatment assignment. Calibration was repeated 5 times on recession patients and on photographs. At each calibration session recession depth, KTW, EHI and PD were evaluated. Calibration was accepted if more than 90% of the measurements could be reproduced within a 1 mm- difference.

### Sample size

The null hypothesis was that there is no difference in the early wound healing index regarding all parameters. The alternative hypothesis states that there is a median difference of at least 1 unit between the active test group and the placebo group. Due to lack of previous RCTs investigating CoQ10 after recession coverage surgery this RCT has to be considered as pilot study.

### Trial monitoring

An independent study monitor from the clinical trial center of the University of Bern conducted the study monitoring checking protocol issues, patient recruitment and enrollment, randomization, intervention and data collection.

### Statistical analysis

Data analyses were performed using a specialized software (Prism v7 (GraphPad Software, La Jolla, CA, USA). The clinical parameters were tested for group differences and depicted as means and standard deviation in the tables. In order to test whether the variables are normally distributed, the data were graphically visualized as QQ plots and examined by the Shapiro Wilk test. As both isolated and multiple recession defects were included, the data were analyzed on patient-level. In case of multiple recessions means were calculated. Kruskal-Wallis test was performed for intergroup differences. A *p* value of 0.05 was set for statistical significance.

## Results

Thirty-four participants were included (25 women and 9 men) and randomised. Four patients (2 out of each group) were lost, of whom one was erroneously operated with enamel matrix derivative, two did not attend the visit at day 21 and one patient withdrew from the study at day-2-visit. This resulted in 30 participants (23 (77%) women and 7 (23%) men) meeting all inclusion criteria and attending all visits. The age ranged from 24 to 50 years with a mean age of 32.58 ± 7.84 years. The patient demographics and characteristics are displayed in Table 1. The groups exhibited no differences concerning the baseline characteristics. Probing depth (PD) was statistically significantly higher in the test group compared to the placebo group (1.82 ± 0.59 mm vs 1.38±0.42 mm; p=0.0457). On average subjects allocated to the test group had 1.8±0.7 teeth/per person and the placebo group 2.3±1.2 teeth (p=0.307).

**Table 1:** Patient characteristics

Test group participants	Gender	Age in years	Smoking status	Recession type defect	Tooth number
1	f	30	n	1	35, 36
4	f	26	n	2	41
5	f	37	n	1	36
14	m	35	n	1	13, 14
15	f	27	n	1	34, 35, 36
16	f	28	n	1	31, 41
19	f	25	n	1	31
21	f	29	n	1	22, 23
25	f	27	n	1	31
27	m	43	n	1	43, 44, 45
29	m	29	n	2	23, 24
30	f	34	n	2	41, 31
31	f	32	n	2	31, 41, 42
32	f	42	n	2	32
33	m	24	n	2	41
Means/total	11 f, 4 m	31.2±5.6	-	1.4±0.4	27 teeth
Placebo group participants	Gender	Age in years	Smoking status	Recession type defect	Tooth number
2	f	33	n	1	11, 21, 22, 23
3	m	31	n	2	43, 42, 41, 32, 33
6	f	44	n	1	13
7	f	50	n	1	41, 31
8	f	26	n	2	31
10	f	32	n	2	41, 31
11	f	30	n	2	42, 41, 31
17	f	42	n	1	41
18	m	25	n	1	31, 33, 34
20	f	37	n	2	42, 41, 31
22	f	50	n	1	13, 15
23	m	18	n	1	31
24	f	36	n	2	41, 31, 32, 33
28	f	23	n	2	31
34	f	30	n	2	41, 31
Means/total	12 f, 3 m	33.8±9.4	-	1.5±0.4	35 teeth

Nine patients of the test and 10 patients of the placebo group had multiple recessions. Six patients in the test and 8 in the placebo group presented with RT2 defects. Baseline recession depth amounted to 3.66 ± 1.43 mm in the test and to 3.49 ± 1.27 mm in the placebo group (p=0.828).

During the healing phase, five patients (3 out of the test and 2 of the placebo group) received antibiotics mostly because of persistent severe pain, severe edema, and malaise.

### Early wound healing

**Fig. 2:**
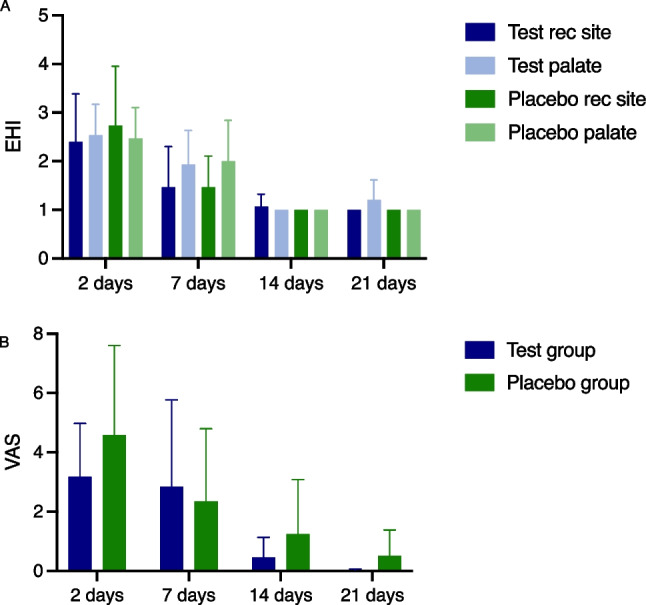
**A**) EHI (early wound healing index) for test recession site, test palatal site, placebo recession site, and placebo palatal site. **B**) VAS (Visual analogue scale) for test and placebo groups for the respective time points. Differences between test and placebo groups were tested using the Mann-Whitney U test. Significance was set at p<0.05 and is marked with *

In terms of early wound healing no clinical differences could be discerned between the groups for any timepoint as assessed by the wound healing index (Table 2). At day 2 the EHI for the recession site amounted to 2.40 ± 0.98 for the test and 2.73 ± 1.22 for the placebo group and dropped to 1.46 for both groups at day 7. The EHI scores for the palate were slightly higher but with no intergroup differences. A subgroup analysis for mandibular and maxillary recessions failed also to reveal any statistically significant differences (Fig. 2A).

**Table 2:** Evaluation of early wound healing and patient-reported pain outcome during the first 21 days postoperatively

Days post-operatively	EHI Recession site		EHI Palatal site		VAS	
	Test	Placebo	Test	Placebo	Test	Placebo
2	2.40±0.98	2.73±1.22	2.53±0.63	2.46±0.63	31.8±17.9	45.8±30.1
P value	0.476		>0.999		0.190	
7	1.46±0.83	1.46±0.63	1.93±0.70	2.0±0.84	28.4±29.3	23.4±24.5
P value	>0.999		>0.999		0.797	
14	1.06±0.25	1.00±0.00	1.20±0.41	1.06±0.25	4.5±6.8	12.4±18.4
P value	>0.999		0.625		0.537	
21	1.0±0.0	1.00±0.00	1.00±0.00	1.00±0.00	0.2±0.4	5.0±8.7
P value	>0.999		>0.999		0.198

#### Patient-reported pain outcome

VAS scores were assessed at day 2, 7, 14, and 21 postoperatively (Table 2). For the test group, VAS scores amounted to 31.8 ± 17.9 mm after 2 days, and thereafter dropped to 28.4 ± 29.3 mm (7 days), 4.5 ± 6.8 mm (14 days) and finally 0.2 ± 0.4 mm (21 days). Similarly, in the control group VAS scores amounted to 45.8 ± 30.1 mm after 2 days, and thereafter dropped to 23.4 ± 24.5 mm (7 days), 12.4 ± 18.4 mm (14 days), and 5.0 ± 8.7 mm (21 days).

The intergroup comparison exhibited no statistically significant differences at any timepoint. However, when comparing the time to recovery, defined as a VAS < 10 mm, then the subjects allocated to the test group reached this threshold after 14 days while the subjects on placebo not until after 21 days (Fig. 2B).

#### Clinical parameters after 6 months

**Fig. 3:**
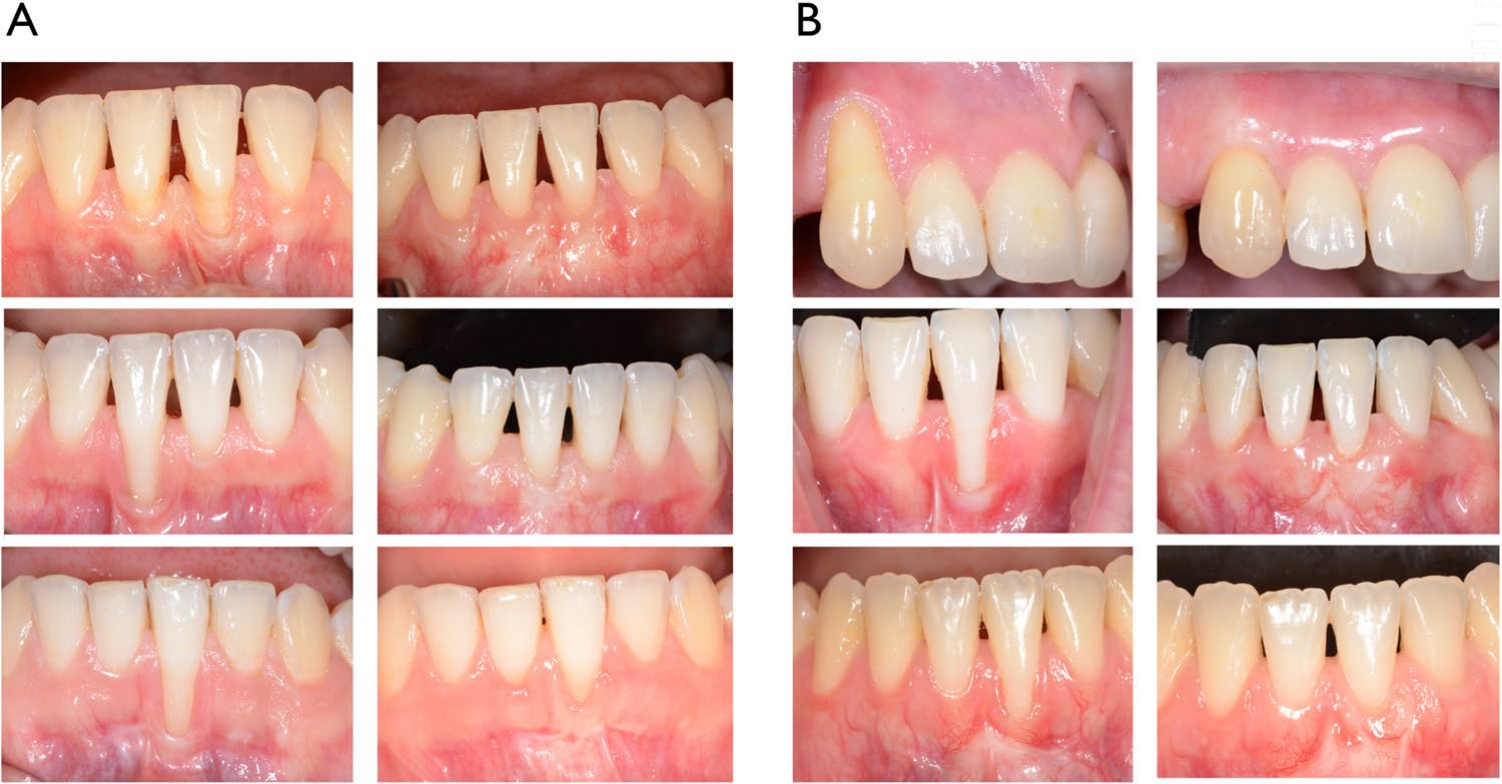
Patients out of test group (**A**) before (left row) and 6 months after (right row) recession coverage surgery. Patients out of placebo group (**B**) before (left row) and 6 months after (right row) recession coverage surgery

**Fig. 4:**
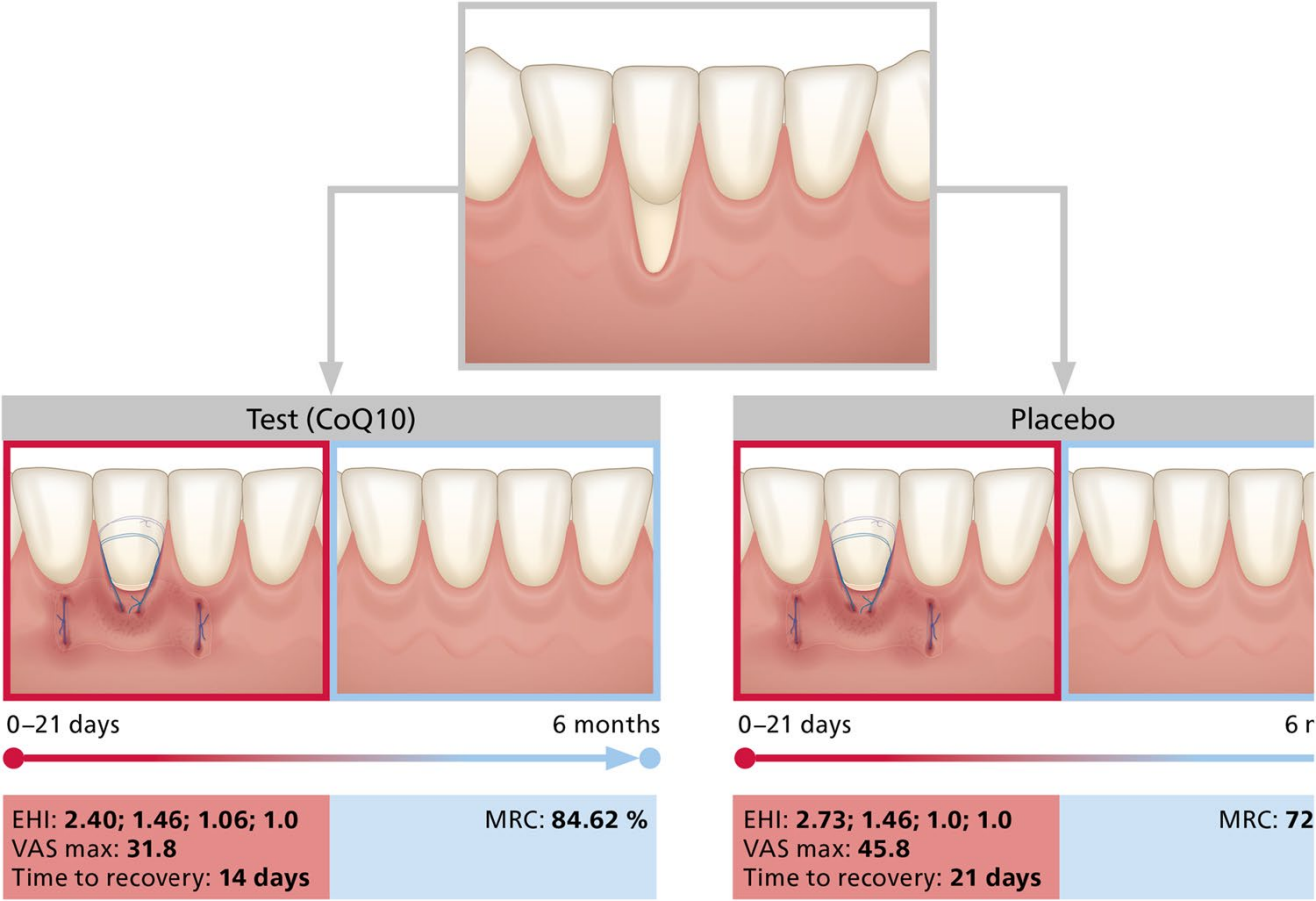
Graphical abstract

No statistically significant differences were observed between the groups for any parameter (Fig. 3A and B). However, there was a trend towards a higher mean root coverage (mRC) in the test group compared to the placebo group (84.62 ± 26.57% versus 72.19 ± 26.30, p=0.0521) (Table 3). Complete root coverage (CRC = 100%) was measured in 9 patients (60.0%) of the test and in 2 (13.3%) of the placebo group. On tooth level CRC was obtained in 17 out of 27 teeth (62.9%) in the test group and in 9 out of 35 teeth (25.7%) in the placebo group. In the test group keratinized tissue width (KTW) increased by 1.66 ± 1.41 mm, similarly in the placebo by 1.00 ± 1.05 mm. The recession depth reduction (ΔRD) was similar in both groups (2.94 ± 1.72 mm vs 2.53 ± 1.54 mm). The esthetic outcomes evaluated by the recession coverage index were as well similar for both groups (Fig. 4).

**Table 3:** Clinical outcomes at the baseline and 6-month visit

Outcome	Test group		Placebo group	
	Baseline	6 months	Baseline	6 months
Rec depth (mean±SD) (mm)	3.66±1.43	0.72±0.99	3.49±1.27	0.96±0.82
KTW (mean±SD) (mm)	2.01±1.13	3.64±1.52	2.05±1.42	3.20±1.33
mRC (mean±SD) (%)		84.62±26.57		72.19±26.30
CRC (number of subjects/%)		9(60.00)		2(13.3)
CRC (number of teeth/%)		17(62.9)		9(25.7)
Δ rec depth (mean±SD) (mm)		2.94±1.72		2.53±1.54
KTW gain (mean±SD) (mm)		1.66±1.41		1.00±1.05
PD (mean±SD) (mm)	1.82±0.59*	1.58±0.72	1.38±0.42	1.42±0.57
Final RES (mean±SD) (score)		7.17±2.10		6.95±1.62

Abbreviations: Rec depth, recession depth; KTW, keratinized tissue width; mRC, mean root coverage; CRC, complete root coverage, RES, root coverage aesthetic score. * Statistical significance compared to the placebo group based on a p<0.05 threshold

## Discussion

CoQ10 application during early wound healing after recession coverage yielded no statistically significant differences concerning clinical healing characteristics and patient-reported pain outcomes between the test and placebo group. Although CoQ10 has favorable properties for wound healing such as anti-inflammatory and anti-oxidative activity, these did not translate into a clinically detectable benefit during the first 21 days of healing. At the 6-month follow-up a trend was observed for mean root coverage being higher in the test group compared to the placebo group (84.62% vs 72.19%). This tendency is further strengthened in the percentage of complete root coverage (CRR).

This trend concerning mean root coverage nonetheless suggests that CoQ10 might have some potential during early wound healing. This is in line with an RCT that has recently evaluated the effect of a CoQ10-collagen-hydrogel on alveolar bone regeneration after tooth extraction in diabetic type II patients. Based on radiographic and histologic evaluation after 3 months, the CoQ10-collagen-hydrogel demonstrated significantly higher bone density and less fibrous tissue compared to the control group [27]. Similarly, rats on long-standing CoQ10 supplementation presented with less age-related alveolar bone loss and a higher bone mineral density while intriguingly, no effect on periodontal tissues was found [28]. When looking at other studies related to CoQ10 and early oral wound healing evidence is yet scarce. In rats, wound healing after tooth extraction was statistically significantly accelerated when CoQ10 encapsulated in nanoliposomes were given, and myeloperoxidase activity as wells as NO concentrations, both responding to oxidative stress, were statistically significantly reduced compared to control [29].

Important to know is that CoQ10 levels significantly decrease with age due to a declining CoQ10 biosynthesis that peaks at around 20 years of age while inversely, oxidative stress is increasing with the years [30, 31]. Decreasing CoQ10 plasma levels were further detected in the course of diseases such as liver cirrhosis or cardiomyopathies. The patient cohort of the present study, however, was young and super-healthy (healthy user effect) with a mean age of 32 years and thus presumably still high levels of endogenously produced CoQ10. Consequently, it might be speculated whether patients of older ages or suffering from chronic inflammatory diseases such as periodontitis/peri-implantitis might benefit more from CoQ10 application or supplementation. This assumption is strengthened when looking at CoQ10 effects in a broader context. Animal studies suggest that CoQ10 supplementation is able to counteract tissue alterations such as cardiac tissue damage that occur following unhealthy high-fat diets whereas no beneficial effects were observed for animals in healthy conditions [18, 32]. These preclinical results were confirmed in clinical studies. While patients presenting with cardiovascular diseases responded well to CoQ10 supplementation i.e., decreased mortality [33], improved ejection fraction, endothelial function and slowing down of the atherosclerotic process [34–36], healthy participants demonstrated no improvement in their endothelial flow-mediated dilatation [37]. Therefore, further well-designed RCTs with CoQ10 are warranted in an elderly patient segment and with chronic inflammatory disease such as periodontitis.

Here, the EHI in the palate was 2.40 ± 0.98 for the test and 2.73 ± 1.22 for the placebo group at day 2 post-surgically. This is in line with the mean EHI after one week reported by Fickl et al. [38]. In terms of palatal wound healing, though, post-surgical flap thickness might be decisive for EHI [39]. Further, the use of platelet rich fibrin has been shown to lower the EHI [40].

In the present study, patient-reported pain outcomes did not differ between the groups at any time-point. The mean VAS scores for both groups were within the range of a previous paper of our group [41]. Recently, Tavelli et al. reported on VAS scores within the first five days spiking at 24.2 to 36.4 mm [42]. Compared to our results (31.8 and 45.8 mm) their values were lower. This might be partly explainable by the fact that they did not create a second wound on the palate by using a volume stable matrix. Further they had slightly different follow-up time points and prescribed antibiotics for 7 days (i.e., 500 mg Amoxicillin tid). Consequently, their groups reached the recovery timepoint (VAS<10 mm) between 8 to 11 days while in the present study at day 14 to 21.

Mean root coverage amounted to 84.64% for the test and 72.19% for the placebo group and showed a trend in favor of the test group. One could argue that this might be caused by the slightly higher number of involved teeth/patients in the placebo group and slightly more RT2 defects in this group. When, however, subgrouping the patients on placebo with the highest number of teeth involved (i.e., patient number 2, 3 and 24) they reached a mRC above the group mean (i.e.,73.69% together, two of them RT2 defects). On the other hand, the patients who performed worst in the placebo group both exhibited RT1 defects, one with maxillary recessions and the other one with an isolated mandibular recession (patient number 22 and 23, 43.75% and 0% mRC). The same was true for the test group of which the patients with the least mRC (50% and 0%, i.e., patient number 15, 19) both presented RT1 defects.

Tavelli et al. obtained 88.25% mRC for the subjects allocated to collagen matrix plus platelet derived growth factor (PDGF) and 77.72% for the subjects allocated to collagen matrix alone. These slightly higher values compared to the present study might be related to differences in inclusion criteria, surgical protocol, and evaluation procedures–Tavelli et al. for example only included RT1 defects while this study involved at half RT2 defects and greater initial recession depths. In a previous RCT of our group mRC values of 77% and 78% were obtained after 6 months [41]. Very high mRC of 91-95% and 93.7% following MCAT and CTG were recently reported by Gorski et al. [43] and by a case series of Aroca et al. [23].

At 6 months, gain of mean keratinized tissue width (KTW) measured 1.66 mm in the test and 1.00 mm in the control group, respectively. In the literature KTW gain varies considerably from 0.32 mm for a collagen matrix and PDGF to 2.0 mm and more [42, 44, 45]. Consequently, CoQ10 had no influence on KTW gain.

Mean root coverage esthetic score (RES) measured 7.17 and 6.95 points for test and placebo group, respectively. When we compare these results with those from the literature, Tavelli et al. evaluated a mean score of 6.98 and 8.17 points, Gorski et al. 8.9 and 8.7 [46] for MCAT and SCTG with or without EDTA, while Stefanini et al. have reported 7.85 and 7.34 for CAF with and without a collagen matrix [47]. When using hyaluronic acid together with MCAT and connective tissue grafts our group previously evaluated a slightly higher score of 7.9 ± 1.9 [48].

This study has some limitations. The early wound healing index was originally established for periodontal surgery. The tunnel flap though mirrors not exactly the situation the index was created for by Wachtel [24]. Many other healing indices have been used in studies which makes comparisons difficult. Notwithstanding, all of these indices are soft tools, that lie in the clinician‘s eyes. Further insights into wound healing would have been gained by assessing biological markers in the gingival crevicular fluid (GCF) during the early wound healing such as for example the level of reactive oxidative species (ROS), transforming growth factor (TGF)- β, inflammatory markers like interleukin (IL) -1β, TNF-α, or the activity of anti-oxidant enzymes superoxide dismutase (SOD) or catalase. A recent split-mouth RCT assessed the effect of enamel matrix derivative in recession coverage and evaluated the main inflammatory markers and growth factors (IL-1β, IL-6, IL-8, fibroblast growth factor (FGF), macrophage inflammatory protein (MIP)-1α and β, platelet-derived growth factor (PDGF), TNF-α, and vascular endothelial growth factor (VEGF)) [49]. Only VEGF yielded significant differences at 7 and 14 days postoperatively.

Finally, the recession defects were not standardized, both multiple and single recessions, RT 1 and 2 in mandible and maxilla were included. In the placebo group slightly more teeth were involved than in the test group (35 versus 27) and 8 versus 6 RT 2 defects. This has to be taken into account when judging the tendency of an increased mRC after 6 months.

To the best of our knowledge this is the first RCT that investigated the effect of CoQ10 on early wound healing and on 6-month results after recession coverage. Our results suggest that during the early phase of wound healing no differences were clinically detectable in this patient groups, CoQ10 had no significant influence on the healing pattern and 6 months after surgery mRC were similar for both groups, albeit with a tendency for a higher mRC in the test group. For future studies it would be more sensible to use CoQ10 in patients of increased age or with inflammatory diseases to profit more given declined endogenous CoQ10 levels and increased oxidative stress.

### Electronic Supplementary Material

Below is the link to the electronic supplementary material.

Supplementary file1 (DOTX 58 KB)
