# Supplementary material for: Effect of Coenzyme Q10 on early wound healing after recession coverage surgery with the modified coronally advanced tunnel technique and a connective tissue graft: A 6-month, triple-blinded, randomized, placebo-controlled pilot trial
Source: Clin Oral Investig. 2024 Jul 11;28(8):424. doi: 10.1007/s00784-024-05790-4 (PMC11239743; doi:10.1007/s00784-024-05790-4)
Supplement: Supplementary file 1 — Supplementary file1 (DOTX 58 KB) [file 784_2024_5790_MOESM1_ESM.dotx]

Figure 4. CONSORT flow diagram.


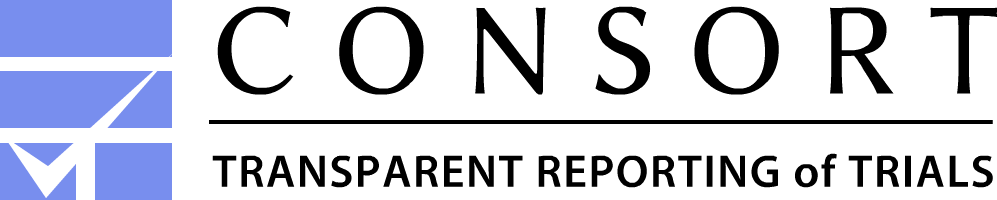


**CONSORT 2010 Flow Diagram**

## Follow-Up

Analysed (n=15)
♦ Excluded from analysis (n=0)

## Analysis

Analysed (n=15)
♦ Excluded from analysis (n=0)

Lost to follow-up (did not come to visit at day 21) (n= 1)

Lost to follow-up (1 did not come to visit at day 21 and 1 withdrew from the study) (n=2)

## Enrollment

Allocated to intervention (n=18)

♦ Received allocated intervention (n=17)

♦ Did not receive allocated intervention (was falsely treated with enamel matrix derivative) (n=1)

## Allocation

Allocated to intervention (n=16)

♦ Received allocated intervention (n=16)

♦ Did not receive allocated intervention (n=0)

Randomized (n=34)

Excluded (n=2)

♦  Not meeting inclusion criteria (n=2)

Assessed for eligibility (n=36)

$
